# Chiropractic approach to the management of children

**DOI:** 10.1186/1746-1340-18-16

**Published:** 2010-06-02

**Authors:** Sharon A Vallone, Joyce Miller, Annica Larsdotter, Jennifer Barham-Floreani

**Affiliations:** 1 Private Practice, Connecticut, USA; 2 Kentuckiana Children's Center, Louisville, KY, USA; 3 Post Graduate Faculty, International College of Chiropractic Pediatrics, Arlington, VA 22201, USA; 4 Lead Tutor MSc Advanced Practice Paediatrics, Bournemouth University, UK; 5 Private Practice, Sydney, Australia; 6 Private Practice, Melbourne, Victoria, Australia

## Abstract

**Background:**

Chiropractic (Greek: done by hand) is a health care profession concerned with the diagnosis, treatment and prevention of disorders of the neuromusculoskeletal system and the effects of these disorders on general health. There is an emphasis on manual techniques, including joint adjustment and/or manipulation, with a particular focus on joint subluxation (World Health Organization 2005) or mechanical lesion and restoring function. The chiropractor's role in wellness care, prevention and treatment of injury or illness is based on education in anatomy and physiology, nutrition, exercise and healthy lifestyle counseling as well as referral to other health practitioners. Depending on education, geographic location, scope of practice, as well as consumer preference, chiropractors may assume the role of primary care for families who are pursuing a more natural and holistic approach to health care for their families.

**Objective:**

To present a perspective on current management of the paediatric patient by members of the chiropractic profession and to make recommendations as to how the profession can safely and effectively manage the paediatric patient.

**Discussion:**

The chiropractic profession holds the responsibility of ethical and safe practice and requires the cultivation and mastery of both an academic foundation and clinical expertise that distinguishes chiropractic from other disciplines.

Research into the effectiveness of chiropractic care for paediatric patients has lagged behind that of adult care, but this is being addressed through educational programs where research is now being incorporated into academic tracks to attain advanced chiropractic degrees.

**Conclusion:**

Studies in the United States show that over the last several decades, chiropractors are the most common complementary and alternative medicine providers visited by children and adolescents. Chiropractors continue to seek integration with other healthcare providers to provide the most appropriate care for their paediatric patients.

In the interest of what is best for the paediatric population in the future, collaborative efforts for research into the effectiveness and safety of chiropractic care as an alternative healthcare approach for children should be negotiated and are welcomed.

## Background

Chiropractic (Greek: done by hand) is a health care profession concerned with the diagnosis, treatment and prevention of disorders of the neuromusculoskeletal system and the effects of these disorders on general health. There is an emphasis on manual techniques, including joint adjustment and/or manipulation, with a particular focus on joint subluxation (World Health Organization 2005) or mechanical lesion and restoring function [1]. The chiropractor's role in wellness care, prevention and treatment of injury or illness is based on education in anatomy and physiology, nutrition, exercise and healthy lifestyle counseling as well as referral to other health practitioners. Depending on education, geographic location, scope of practice, as well as consumer preference, chiropractors may assume the role of primary care for families who are pursuing a more natural and holistic approach to healthcare for their families [2]. In this role, they may also provide "well child" care, monitoring growth and development.

The purpose of this paper is to present a perspective on current management of the paediatric patient by members of the chiropractic profession and to make recommendations as to how the profession can safely and effectively manage the paediatric patient.

## Discussion

### Use of Chiropractic by Children

According to a report published in 2000 by Lee, Li and Kemper, the number of children visiting chiropractors was substantial and increasing [3]. A 2007 study by National Center for Health Statistics showed that the most common provider-based complementary and alternative therapy used by children in the United States was chiropractic or osteopathic manipulation [4]. Other recent studies in the United States show that approximately14% of chiropractic patients are children under 18, and that chiropractors are the most common complementary and alternative medicine (CAM) providers visited by children and adolescents [5]. In 2007, Jean and Cyr, in a survey of paediatric patients in an outpatient facility, found that 19% of the families sought chiropractic care for their children [6]. Carlton, Johnson and Cunliffe reported on the factors influencing parents' decisions to choose chiropractic care by surveying families with children ages 5-11 years in a typical county in the United Kingdom. The results indicated that parents who already used chiropractors were more likely to take their children to the chiropractor, but that the overall utilization of CAM was most influenced by family physician and friends [7].

### Chiropractic Education in Pediatrics

Chiropractic college coursework has included paediatrics for the last several decades. In 1998, Coulter stated that the average hours of education in US chiropractic colleges assessed was 15 hours for paediatrics [8] in the total chiropractic curriculum which includes a minimum of 4,200 hours of classroom, laboratory and clinical experience [9,10].

All chiropractic colleges' undergraduate courses in paediatrics recognize the unique anatomy and physiology of the paediatric patient. In turn, they promote the understanding that modification of evaluation and therapeutic techniques is required, thus preparing graduating chiropractors to work with their patient from birth through end of life. Chiropractic clinical education prepares the student to assess and manage (or co-manage as appropriate) the paediatric patient with a musculoskeletal problem.

As the profession grew, specialty interest groups were founded amongst national associations in the US (International Chiropractors Association, ICA, and American Chiropractic Association, ACA) as well as by private individuals [11-13]. Postgraduate education became available in both private entrepreneurial and academic venues. Academic venues offered by or sponsored by chiropractic colleges included individual postgraduate educational seminars and certification courses of approximately 100 to 120 hours. One such certification has, in the past, been offered by both the ICA Council on Chiropractic and the Anglo European Chiropractic College. Currently, this one-year certification program continues to be offered by the privately held International Chiropractic Paediatric Association (ICPA).

This one year certificate program may serve as the first year of study of the more advanced three year programs that confer diplomate status. For example, the International College of Clinical Chiropractic's program [11] offered in conjunction with the post graduate departments of chiropractic colleges like Palmer College of Chiropractic, New York College of Chiropractic and the New Zealand College of Chiropractic, consists of 360 classroom hours and includes required papers and annual exams before the candidates are eligible to sit for the board examination to qualify them for the Diplomate in Clinical Chiropractic Pediatrics. The International Chiropractic Paediatric Association (ICPA) also offers a diplomate program and testing is administered through the Academy of Chiropractic Family Practice [14].

In the European Union, there are currently two institutions offering a Masters in Science (MSc) with a specialty in paediatrics. These are AngloEuropean College of Chiropractic in conjunction with Bournemouth University and McTimoney Chiropractic College in conjunction with the University of Wales [15,16].

### What Types of Cases Present to the Chiropractor?

The age range of paediatric patients visiting chiropractic clinics ranges from premature infants to adolescents. Besides those conditions traditionally classified as musculoskeletal (for example, torticollis, scoliosis, sprain/strains and spinal pain), there are also musculoskeletal presentations that include a somatovisceral component including, but not limited to, persistent crying and feeding problems in infants (like difficulty breastfeeding, colic), sleep disruption, otitis media, enuresis, asthma, headaches, constipation, learning disorders and a variety of presentations on the autistic spectrum [17,18].

### What is Chiropractic Management?

Chiropractors should obtain a full history and perform a complete, age appropriate examination, based on the presenting clinical symptoms as well as the general condition of the patient (Appendix 1). Depending on the circumstances, a written and/or an oral interview about the chief complaint, its history and a survey of systems may be completed, as well as performing an exam which may be comprehensive or regional, with a more detailed follow up after the acute situation has been assessed and addressed. A comprehensive description of this complete intake and examination is beyond the scope of this paper. But it is important to emphasize that the clinician must carefully discern whether an infant is ill by physical examination (including temperature, pulse, respiration rate and effort, pallor, muscle tone and irritability or lethargy) and observation of any of the signs of illness of infancy (Table 1). Appropriate referral for co-treatment or alternative treatment should be made. Additionally, growth should be plotted and interpreted by using growth charts [19]. There should also be clear evidence that there are no red flags prior to accepting a paediatric case.

**Table 1:** Serious Signs and Symptoms of Children that Require Immediate Medical Referral

Symptom/Sign	Explanation/Implication
**Neonate**	Since the health status of a neonate can change rapidly, any signs of illness require immediate referral.

**Lethargy**	Absence of interaction, hypotonia and/or crying

**High Respiratory Rate**	Rapid or difficult respirations not related to activity; respiration rate >60 breaths/minute with rib recession

**Blue Lips or Tongue**	May indicate reduced blood oxygen level

**Dehydration**	Common sequel to diarrhea or vomiting. Dry mouth, sunken fontanelle, tenting skin, <4 wet nappies/diapers (60-90 mL/4-6 TBS). Urine should be pale and mild smelling.

**Pain and Tenderness**	Child screams when touched or being moved; avoids being held. Sudden onset of groin pain in a boy may be a sign of testicular torsion; episodic screaming in young children may be a sign of intussusception

**Tender Abdomen**	Inability to tolerate 2 cm abdominal impression; bloated or rigid abdomen

**Inability to Walk**	Refusal or inability to walk in child who previously was walking (or crawling); development of a limp requires attention

**Bulging Fontanelle**	Evident bulge and rigidity in anterior fontanelle in a quiet child in an upright position

**Stiff or Rigid Neck**	Refusal/inability to look toward their toes or at a toe placed on their chest may be an early sign of meningitis; very young infants may have meningitis with no obvious signs of neck stiffness

**Petechiae**	Purple or blood-red spots on the skin that do not blanch with pressure may be a sign of bloodstream infection. Exclude bruises that have an explanation

**High Fever**	Referral for consult: Neonates (<28days): ≥38 C (100F); 28-90 days >38 C with signs of toxicity or incessant crying; 91-36 months: >39 C (102.2F) and signs of toxicity [58].

**Drooling**	Sudden onset of drooling not associated with teething, especially when associated with difficult swallowing, may be a sign of epiglottal or pharyngeal infections

Once a child's condition is diagnosed and the condition is considered by the clinician to be potentially responsive to chiropractic care, parental permission is obtained and chiropractic treatment is administered.

An appropriate management plan should be brief and take into account the condition, age and size of the child, and it should be clear that intervention is affecting change ahead of the natural resolution history of the disorder. The clinician should demonstrate a clear understanding of the case, and should communicate with the parent in a manner which allays their anxiety. The clinician should obtain written evidence of receipt of permission to examine and treat the infant or child by a parent who is able to consent. Contra-indications to both treatment and types of treatment are outlined in Table 2.

**Table 2:** Absolute and Relative Contraindications to Manual Therapy

ABSOLUTE CONTRAINDICATIONS	
**Indication**	**Explanation**

**Withdrawal of consent by the parent or child**	Potential for litigation

**Hypermobility of the joints of the child**	Increased flexibility of joint structures and less muscular resistance than the adult

**Long-lever and high force manual procedures**	Anatomically immature: no joint "lockup."

**Occipito-atlantal &** **Atlanto-axial instability**	Common in children with Down Syndrome, Juvenile Rheumatoid Arthritis, Marquio's, Klippel-Feil Syndrome

**Brain or spinal tumors**	Potential of neurologic damage or vascular compromise by the introduction of specific or non-specific force due to the pathophysiology or anatomical position of the tumor; immediate referral to appropriate healthcare provider

**Active metaphyseal** **growth tissue**	Zone of provisional calcification- the transitional region between cartilage and newly formed metaphyseal bone is subject to separation and avascular necrosis when subject to force

**RELATIVE CONTRAINDICATIONS/Need for caution**	

**Cervical Spine adjustments**	Reduce the incidence of potential adverse event by refraining from over treating the sensitive structures of the cervical spine

**Down Syndrome or other congenital anomalies**	If you see an anomaly in one region, be suspicious of anomalies elsewhere.

**Recent upper respiratory tract virus**	Potential for inflammatory disruption to the atlanto-axial joint

**Symptoms and signs incongruous with** **palpatory findings.**	Diagnosis requires corroboration of signs and symptoms with exam findings (including palpatory findings). When they are incongruous, further diagnostic studies should be ordered to rule out any potentially serious underlying pathology.

**History of sleep-disorder in infants <12 weeks of age**	Watchful waiting first 12 weeks (rule out Arnold Chiari Syndrome)

**Inversion of neonate or young infant**	Relative contraindication secondary to neonatal circulation and clotting factors, respiratory distress, cranial and cervical birth trauma, undiagnosed perinatal or postnatal stroke, undiagnosed hip dysplasia.

Chiropractic management of paediatric patients may include advice about nutrition and exercise, in-clinic rehabilitation procedures, age appropriate paediatric manipulation (modified from adult procedures based on paediatric anatomy) and soft tissue techniques and/or referral to another health provider.

### What type of response to care is typical?

If a chiropractor determines that a mechanical lesion is responsible for the child's symptoms, chiropractors typically address this with manual therapy. Based on the authors' experience, symptoms of this nature would be expected to respond within approximately three to six treatments, depending on the duration of the problem.

After infancy, functional problems are more easily diagnosed with close observation as well as verbal and physical clues from the patient and the parents. Parents should report notable and significant improvement after a few treatments with full recovery shortly thereafter in routine cases. Long-term, complex, and difficult cases would typically require longer-term care and the potential for additional treatments or co-management with other healthcare professionals.

Children with physical or neurologic disabilities may also require more extensive treatment. Often, as demonstrated anecdotally in the academic clinical setting or over the years at facilities like Kentuckiana Children's Center in Louisville, Kentucky, United States [20], when chiropractic treatment is provided in collaboration with other healthcare professionals (nutritionists, occupational therapists, physical therapists, art and recreational therapists, etc.), many children demonstrate improved development or a more consistent maintenance of their quality of life.

### Communication and collaboration benefits patients, healthcare providers and overburdened health care systems

In reviewing the literature over the last decade, CAM health care providers, including chiropractors, have made sufficient inroads into paediatric healthcare to warrant the scrutiny of leaders in the field of conventional western medicine. Published papers explore everything about CAM from the economics [21-24] and utilization [25-31], to review of effectiveness for specific conditions [32-37] as well as ethics, policy and malpractice risks [38,39]. Communication between parent and healthcare practitioner is a particular concern expressed in a number of these scholarly papers. Providers report that parents often fail to communicate that their children are receiving CAM therapies when they visit their offices for routine wellness visits (visits where the GP or pediatrician monitors normal growth and development, administers required immunizations, etc), when the child presents in an emergency room in crisis or when the child is receiving ongoing care for chronic illnesses. It is unknown whether this failure to communicate is due to fear of the provider's censure, a failure to realize significance of the information to the healthcare provider or an unintentional omission. This may be perceived as increasing the malpractice risk for the practitioner when the clinician is administering therapeutic measures without being fully informed. One author suggests that collaborative care in a hospital setting might improve outcome in cases of co- management [40].

### Where is the evidence?

Careful scrutiny of the evidence for the efficacious treatment of a variety of common pediatric complaints demonstrates the need for more research in all fields. One of the most common afflictions of infants, excessive crying or infant colic, serves as an example of the paucity of evidence. Traditional Western medicine has failed to provide any safe, effective therapy for infant colic [41] or for other common complaints of infancy, such as the excessive crying, poor sleep habits (difficulty going to sleep and staying asleep) and sub-optimal feeding. The available evidence is limited about chiropractic therapy for any of these conditions [23]. However, there is also a lack of evidence about any other therapy for these conditions [42,43].

There is some evidence that taking a colicky infant to a chiropractor will result in decreased crying [44]; it is not known whether this response is specific to paediatric manipulation or whether there may be multiple non-specific effects at play [19,45-47]. Currently two randomized trials, one in England and one in Denmark, are in process now to gather more definitive evidence on this issue. This evidence as it exists can be made available to the parents. It can also be explained that the recommendation for care made by the chiropractor is based on, not only the available evidence, but also his or her professional experience coupled with the low risks of adverse effects [48,49]. This makes a therapeutic trial of chiropractic care for infantile colic a viable alternative for the parents to consider when evaluating the full picture of available, effective treatments.

It is also imperative that in this same context of informed consent, the treating chiropractor must qualify him- or herself to the parents/patient as having mastered appropriate skills and fully evaluated the child (as outlined earlier), ruled out contraindications to chiropractic care and have made appropriate referrals before, or in addition to, providing chiropractic treatment.

As stated earlier, negative side effects of paediatric manipulation are rare and mild [48,49]. The risks of harm (from potential child abuse) coming to an inconsolable crying baby without intervention can be significant [50,51]. We therefore estimate that the risk/benefit ratio falls into the camp of a short (two week) trial of chiropractic treatment until and unless evidence accumulates to show no effect of such treatment. Additional research should investigate whether this therapeutic contact with the chiropractor may have provided a safe haven for these families to vent the frustration and difficulties of dealing with a crying baby, reducing the risk of injury to the infant by a frustrated parent.

### How safe is manual therapy for the paediatric patient?

Although manual therapy is the treatment identified with chiropractors, chiropractic is a profession, not a therapy. Manual therapy is provided by many other types of clinicians including osteopaths, medical doctors, physiotherapists, cranial sacral therapists and multiple alternative practitioners. This creates a problem when reviewing the safety record of manual therapy. A recent systematic review of the safety record of manual therapy for paediatric patients showed that there were 14 reported adverse events in 41 years [44]. Nearly half of those injuries were caused by non-chiropractic clinicians who represent a small minority of those performing manual therapy. In fact, chiropractors provide 94% of manipulative care in the United States [52]. Any adverse event should be avoided, but any treatment able to effect positive change may put the patient at some risk. The evidence, so far, is that manual therapy for the paediatric patient, in the hands of a skilled chiropractor, has a very low risk.

Chiropractors are committed to gathering all data relative to risks. In the United Kingdom, there is a profession-wide initiative called the Chiropractic Patient Incident Reporting and Learning System http://www.CPiRLS.org. This is an on-line forum on which chiropractors share all patient safety incidents, including paediatrics. It is used by all registered chiropractors in the United Kingdom. There is a similar system in place in Switzerland as well. The system for the European Chiropractic Union is currently in the guidelines stage. Prospective monitoring of all safety incidents is the way forward to track risks to treatment of the paediatric patient.

### Ethics and responsibility as practitioners

Recently, a multidisciplinary panel of chiropractors was able to reach consensus regarding the chiropractic approach to the paediatric chiropractic patient "based on both scientific evidence and clinical experience". This demonstrated an effort on the part of the profession to establish standards to guide practising clinicians [53].

Research into the effectiveness of chiropractic care for paediatric patients has lagged behind that of adult care, but this is being addressed through educational programs where research is now being incorporated into academic tracks to attain advanced chiropractic degrees.

The responsibility of ethical and safe practice lies within the profession. This begins with an acknowledgement that it requires the cultivation and mastery of both an academic foundation and clinical expertise in the art, science and philosophy of chiropractic to distinguish the chiropractic profession from other disciplines. Chiropractic is a profession, not a technique and chiropractors are responsible for diagnosis and appropriate management of any case they accept.

For example, determining the necessity of care for the paediatric population is not necessarily justified by the usual criteria of specific objective measurements such as a level of impairment, pain or range of motion. The paediatric patient may be evaluated utilizing these traditional criteria but may also have other objective findings that support the necessity for chiropractic care like the presence or absence of infant reflexes or relative attainment of developmental milestones secondary to neurologic or motor impairment (feeding, sitting, crawling, etc). Therefore an understanding of child development is critical for treatment of a pediatric patient. If a clinician is not appropriately trained in evaluating or treating a child, then becoming acquainted with colleagues who are competent is strongly recommended. The chiropractor's responsibility goes beyond the application of chiropractic principles and practice, but also in the timely recognition of critical red flags and the need for referral for collaborative treatment to chiropractors or other appropriate healthcare professionals.

Amassing evidence for the effectiveness and safety of chiropractic care for children is gradually progressing, thanks to the dedication of academicians and clinicians around the world. Authors such as Hawk and Fallon [54,55] have expounded on the challenges we face in attempting to develop ethical, safe and comprehensive models to study. It is important that the problems of infancy and children which cause suffering to children and families and use significant health care and community resources should be high on the list of conditions to investigate.

## Conclusion

Studies in the United States show that over the last several decades, chiropractors are the most common CAM providers visited by children and adolescents. Chiropractors continue to seek integration with other healthcare providers to provide the most appropriate care for their paediatric patients.

In the interest of what is best for the paediatric population in the future, collaborative efforts for research into the effectiveness and safety of chiropractic care as an alternative healthcare approach for children should be negotiated and are welcomed.

## Competing interests

The authors declare that they have no competing interests.

## Authors' contributions

SAV originally conceived of the conceptual basis of the manuscript. SAV and JM shared the writing of the initial manuscript, and this was circulated amongst all authors for editing and revisions until the final manuscript was agreed upon. All authors took part in researching, editing and revising the manuscript on multiple occasions.

## Appendix 1: Chiropractic Assessment of the Pediatric Patient [56,57]

### History and Survey of Symptoms

A focused history of the chief complaint should be age and situation-specific but a complete history (from gestation) should also be obtained. A complete history should include (but is not limited to):

• Parents' health history (including relevant genetic history)

• History of mother's previous and current pregnancies, including ante partum and intra partum events

• Complete intervening health history of patient including illness, accidents, surgeries, hospitalizations, previous chiropractic or other therapies, concurrent diagnosis or intervention for presenting condition and the patient's response to care.

• Survey of systems involves reviewing responses to questions asked in an interview or in a detailed questionnaire which will reveal perceived or actual level of function of organ systems of the body. If using a questionnaire, review questionnaire with the patient, parent(s) or guardian and record in patient notes.

### Assessment

Growth, head circumference and gestational age:

• use age specific World Health Organization growth charts

Vital signs:

• Heart rate (blood pressure if appropriate to evaluate in a particular situation)

• Respiratory rate/temperature/pallor/skin turgor

Physical examination- appropriate for age and presenting clinical symptoms and general health of patient. To include (but not be limited to):

• Visual assessment for gross morphologic changes, discoloration, deformity, atrophy, etc.

• Auscultation chest (heart and lungs) and abdomen

• Palpation: cranium including fontanelles, lymph nodes, soft tissue, abdomen and skeletal structures

• Neurologic and orthopedic examination to include:

a. Reflexes (Infantile and Deep Tendon Reflexes)

b. Range of motion and joint integrity

c. Muscle mass, tone and strength

d. Integrity of sensory system (including sensory processing)

• Age appropriate developmental evaluation

• Large motor skills (ranging from antigravity muscular control to locomotion)

• Small motor skills (manual dexterity with simple and complex skills)

• Language (receptive and expressive)

• Cognition, demeanor and social skills

Chiropractic Assessment:

• Posture (appropriate to developmental age) and alignment of skeletal structures

• Pedal integrity (rule out pes planus, pes cavus, club foot, etc)

• Cranial and skeletal motion

• Soft tissue integrity, restriction, adhesion or fibrosis
